# Associations between vascular risk factors and subsequent Alzheimer’s disease in older adults

**DOI:** 10.1186/s13195-020-00690-7

**Published:** 2020-09-26

**Authors:** Hyewon Lee, Kiwon Kim, Yeong Chan Lee, Soyeon Kim, Hong-Hee Won, Tae Yang Yu, Eun-Mi Lee, Jae Myeong Kang, Matthew Lewis, Doh Kwan Kim, Woojae Myung

**Affiliations:** 1 Department of Health Administration and Management, College of Medical Sciences, Soonchunhyang University, Asan, South Korea; 2 Department of Psychiatry, Veteran Health Service Medical Center, Seoul, South Korea; 3 Samsung Advanced Institute for Health Sciences and Technology (SAIHST), Sungkyunkwan University, Samsung Medical Center, Seoul, South Korea; 4 Division of Endocrinology and Metabolism, Department of Medicine, Wonkwang Medical Center, Wonkwang University School of Medicine, Iksan, South Korea; 5 Department of Health Science, Dongduk Women’s University, Seoul, South Korea; 6 Department of Psychiatry, Gil Medical Center, Gachon University College of Medicine, Incheon, South Korea; 7 The Department of General Practice, Melbourne Medical School, The University of Melbourne, Melbourne, Australia; 8 Department of Psychiatry, Samsung Medical Center, Sungkyunkwan University School of Medicine, Seoul, South Korea; 9 Department of Neuropsychiatry, Seoul National University Bundang Hospital, Seongnam-si, South Korea

**Keywords:** Alzheimer’s disease, Lipids, Blood pressure, Risk factor

## Abstract

**Background:**

The clinical guidelines related to the primary prevention of Alzheimer’s disease (AD) have focused on the management of vascular risk factors. However, the link between vascular risk factors and AD in older adults remains unclear. This study aimed to determine the association between vascular risk factors and subsequent AD in 178,586 older adults (age ≥ 65 years).

**Methods:**

Participants were recruited from 2009 through 2010 and followed up for 6 years. We assessed various vascular risk factors (total cholesterol [TC], low-density lipoprotein cholesterol [LDL-C], high-density lipoprotein cholesterol [HDL-C], triglycerides [TG], fasting glucose [FG], systolic blood pressure [SBP], diastolic blood pressure [DBP], pulse pressure [PP], and body mass index [BMI]) and their association with AD incidence, categorizing each vascular factor using current clinical guidelines.

**Results:**

AD was observed in 6.0% of participants at follow-up. All lipid profiles (TC, LDL-C, HDL-C and TG) were positively associated with the risk of AD. SBP and PP were in negative associations with AD, and DBP was positively associated with AD. BMI exhibited a negative association with AD incidence. We found no significant association between FG and AD risk. The sex difference was observed to have effects on vascular risk factors.

**Conclusions:**

In this study, we comprehensively investigated the association between eight vascular risk factors and the risk of incident AD. Our findings suggest that multiple vascular risk factors are related to the development of AD in older adults. These results can help inform future guidelines for reducing AD risk.

## Background

Alzheimer’s disease (AD) is among the most prevalent neurological disorders, accounting for 10.2% of the global disability-adjusted life year (DALY) [1]. Moreover, researchers have projected that over 60 million individuals worldwide will be diagnosed with AD by the year 2050 [2]. Trials for AD-modifying drugs have yielded disappointing results, increasing the need for primary prevention efforts.

Current guidelines related to the primary prevention of AD focus on the modification of vascular risk factors (e.g., dyslipidemia, blood pressure [BP], fasting glucose [FG], or weight) [3–7]. Vascular risk factors in midlife and late-life, associated with the risk of AD, showed inconsistent profiles, with midlife vascular risk factors increasing the AD risk, contrary to late-life factors [8].

Midlife hypertension has been reported to increase AD dementia risk [9], while late-life hypertension has an inconsistent association with AD dementia risk in the same research. The FG level in midlife was also reported to have an increased association with AD risk which was in line with late-life poor glucose control [10], but this association was not consistent with previous studies [11]. The association between lipid profile in midlife and late-life with AD risk is similar to that of BP. Increased lipid level in midlife was reported to increase AD risk [12], but other researchers reported mixed results with interventions [13, 14]. However, late-life lipid level and AD risk were more complicated, demonstrating either opposite results or no significance [15]. Midlife obesity is an established risk factor for AD, whereas late-life obesity has been proposed as a protective state, exhibiting reverse causation [16]. As it could be supported by these complex reports in midlife and late-life vascular risk factor studies, the preventive effect of modifying vascular risk factors in older adults is still insufficient.

Additionally, the majority of previous studies have examined a limited number of risk factors at a time, and some of these studies have not considered the confounding effects of comorbid illnesses or related modifiable lifestyle factors [17]. The difference on the risk of AD linked to vascular risk factors based on sex is also an emerging issue to be explored. This difference has been related to different factors between male and female including systematic inflammation, metabolic condition, vascular dysfunctions and immobilization [18]. In this nationwide population-based study of older adults, we aimed to investigate the association between multiple vascular risk factors and subsequent AD after comprehensive adjustment for covariates. We hypothesized that the vascular risk factors in older adults could have an association with the risk of AD, concordant with recent guidelines suggested in midlife vascular risk factors and AD.

## Methods

### Data source

Data for this nationwide population-based study of older adults in South Korea were obtained from the National Health Insurance Service-Senior Cohort (NHIS-SC) database. The study was approved by the Institutional Review Board of Seoul National University Bundang Hospital, which waived the requirement for informed consent due to the nature of the study. All data were anonymized to maintain confidentiality.

The NHIS is the universal insurance provider for all of South Korea, launched in 2000 as a single-payer system combining more than 366 medical insurance payers [19]. NHIS collects medical records for all citizens, including data related to healthcare utilization (outpatient visits or admission), prescription medications, national health examination results, and demographics, to construct the National Health Insurance Database (NHID). Among the five national cohorts established by the NHIS, the NHIS-SC includes data from 558,147 older adults taken from the total eligible population (age ≥ 60 years; 5.5 million people in 2002) using a simple random sampling method. The NHIS-SC participants were followed up from January 1, 2002, until December 31, 2015.

### Study design and population

**Fig. 1:**
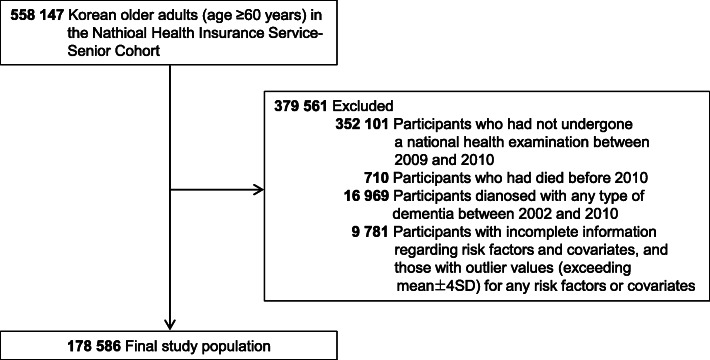
Flow diagram of the study population

We did a nationwide retrospective cohort study using the NHIS-SC data. The assessments of several vascular risk factors (total cholesterol [TC], low-density lipoprotein cholesterol [LDL-C], high-density lipoprotein cholesterol [HDL-C], and triglycerides [TG]) were initiated in 2009. Therefore, we selected a total of 206,046 participants who had undergone a national health examination at least once between 2009 and 2010 from the 558,147 NHIS-SC participants. Then, participants who had died before 2010 (*n* = 710), diagnosed with any type of dementia (*n* = 16,969), with incomplete information regarding risk factors and covariates, and outlier values (exceeding the mean ± 4 standard deviations) for any risk factors or covariates (*n* = 9781) were excluded. The final study population included 178,586 participants (Fig. 1).

### Construction of key variables

Vascular risk factors collected included TC (mg/dL), LDL-C (mg/dL), HDL-C (mg/dL), TG (mg/dL), FG (mg/dL), systolic BP (SBP, mmHg), diastolic BP (DBP, mmHg), and body mass index (BMI, units). We assessed levels of these variables measured during the national health examination in 2009 or 2010. When health examinations were performed in both 2009 and 2010, the levels from 2010 were used. For the main analyses, continuous variables were categorized according to the current Korean guidelines for vascular risk factors [20] and the categorized variables were analyzed as the main variables. The reference levels were < 200 mg/dL for TC, < 100 mg/dL for LDL-C, ≥ 60 mg/dL for HDL-C, < 150 mg/dL for TG, < 70 mg/dL for FG, < 120 mmHg for SBP, < 80 mmHg for DBP, and < 18.5 for BMI. Cutoff values for each category of the vascular risk factors are presented in Additional file 1: Table S1. We also constructed the pulse pressure (PP) variable by subtracting DBP from SBP. PP is considered a surrogate marker for arterial stiffness [21] and known to be an independent predictor for cardiovascular diseases. The PP variable was categorized by its quartile cutoff due to the absence of the referent level for PP, and the first quartile (Q1) was used as a referent level. The quartile cutoffs were 42 mmHg for Q1–Q2, 50 mmHg for Q2–Q3, and 60 mmHg for Q3–Q4.

Incident AD was regarded as the primary outcome. For this study, AD incidence was defined as the first healthcare utilization according to the International Classification of Diseases, Tenth Revision (ICD-10) codes F00 or G30 (primary or secondary diagnosis), accompanied by a documented history of using cognition-enhancing medications (donepezil, rivastigmine, galantamine, or memantine) [22].

### Statistical analysis

We conducted a survival analysis using the Cox proportional hazards regression model, to estimate hazard ratios (HRs) for vascular risk factors associated with subsequent AD incidence. Survival time was calculated from January 1, 2010, to the date of AD incidence, death, or the end of follow-up (December 31, 2015), whichever occurred first. Each vascular risk factor (categorical, see Additional file 1: Table S1) was first evaluated in the age- and sex-adjusted Cox model. Age was included as a continuous variable. The subsequent fully adjusted Cox model included household income (categorical, medical aid, low [1st–3rd decile], middle [4th–7th decile], or high [8th–10th decile]), smoking status (categorical, never, ex-smoker, or current smoker), alcohol consumption (categorical, rarely [0–2 days/week], light [3–4 days/week], or heavy [5–7 days/week]), exercise (categorical, yes or no [1–7 days/week]), hemoglobin (continuous, g/dL), frequency of healthcare utilization (categorical, first, second, third, or fourth quartiles), depression (categorical, yes or no), Charlson Comorbidity Index (continuous), medication history (categorical [yes or no] for each medication category [dyslipidemia medication, antidiabetic medication, antihypertensive medication, antidepressants, benzodiazepines or sleeping aids, and antiplatelet medication]), and all other vascular risk factors (continuous). Household income was estimated based on insurance premiums using NHIS data. The frequency of healthcare utilization, comorbidities, and medication history was calculated based on the data from 2002 to 2010. In the analysis of TC as a major risk factor, LDL-C was excluded in the full model, and in the analysis for PP, DBP was excluded. In analyses for all other vascular risk factors, TC was excluded due to the high correlation between TC and LDL-C. Multicollinearity between all covariates was tested using a variance inflation factor (VIF), which revealed no significant collinearity (VIF < 2 for all variables). *P* values for linear trends were also calculated by treating median values of each categorical group as continuous variables (e.g., 173 mg/dL, 217 mg/dL, and 257 mg/dL for each group of TC). The proportional hazard (PH) assumption was satisfied both graphically and statistically using Schoenfeld residuals. No variables violated the PH assumption.

We examined prescription records for dyslipidemia medication, antidiabetic medication, and antihypertensive medication during the first 3 years of follow-up for all participants, who were then stratified into two groups according to each prescription history. Stratified analyses were performed to investigate whether medication intake during the follow-up period modified the association between vascular risk factors and AD incidence. Analyses were also stratified by sex to assess the potential effect of sex. Significant differences between the two groups were examined using an interaction term between vascular risk factor variables and group indicator variables in the total dataset.

Two-sided analyses were conducted at a significance level of .05, and 95% CIs are reported. SAS Enterprise Guide version 7.2 (SAS Institute Inc) and R Studio version 1.0.136 (RStudio Inc: packages *survival version 2.43-3 and survminer version 0.4.3*) were used to perform the analyses.

## Results

### Characteristics of the study population

During the 6-year follow-up period, 10,732 (6.0%) participants had incident AD. Among them, 94.8% of the patients were prescribed with donepezil, rivastigmine, and galantamine for mild or moderate severity, while the rest of patients (5.2%) were prescribed high doses of donepezil and memantine, which are approved for moderate or severe dementia. Table 1 presents the descriptive characteristics of the study participants in the incident AD and non-AD groups. Female sex, older age, medical assistance, smoking, alcohol consumption, frequent exercise, frequent healthcare utilization, comorbidities (including depression), and a history of medication use were more common in the AD group. Besides, the AD group exhibited higher TC, LDL-C, TG, and FG levels than the non-AD group. Hemoglobin levels and BMI were lower in the AD group than in the non-AD group.

**Table 1:** Descriptive characteristics of the study population

	No. (%) of participants, vascular risk factors			
	Total (***n*** = 178,586)	AD (***n*** = 10,732)	Non-AD (***n*** = 167,854)	***P*** value
Sex*				< 0.0001
Male	81,242 (45.5)	3742 (34.9)	77,500 (46.2)	
Female	97,344 (54.5)	6990 (65.1)	90,354 (53.8)	
Age group*				< 0.0001
65 to 69 years	23,471 (13.1)	74 (0.7)	23,397 (13.9)	
70 to 74 years	80,333 (45.0)	2352 (21.9)	77,981 (46.5)	
75 to 79 years	48,208 (27.0)	3898 (36.3)	44,310 (26.4)	
Older than 80 years	26,574 (14.9)	4408 (41.1)	22,166 (13.2)	
Income, deciles*				< 0.0001
Medical aid	1899 (1.1)	258 (2.4)	1641 (1.0)	
Low (1st–3rd decile)	36,433 (20.4)	2037 (19.0)	34,396 (20.5)	
Middle (4th–7th decile)	48,421 (27.1)	2832 (26.4)	45,589 (27.2)	
High (8th–10th decile)	91,833 (51.4)	5605 (52.2)	86,228 (51.4)	
Lifestyles				
Smoking status*				< 0.0001
Never smoking	130,940 (73.3)	8475 (79.0)	122,465 (73.0)	
Ex-smoking	26,613 (14.9)	1230 (11.5)	25,383 (15.1)	
Current smoking	21,033 (11.8)	1027 (9.6)	20,006 (11.9)	
Alcohol consumption*				< 0.0001
Rarely (0–2 days/week)	158,170 (88.6)	9795 (91.3)	148,375 (88.4)	
Light (3–4 days/week)	9275 (5.2)	376 (3.5)	8899 (5.3)	
Heavy (5–7 days/week)	11,141 (6.2)	561 (5.2)	10,580 (6.3)	
Exercise*				< 0.0001
No exercise	121,405 (68.0)	7795 (72.6)	113,610 (67.7)	
Exercise	57,181 (32.0)	2937 (27.4)	54,244 (32.3)	
Comorbidities				
Depression*	14,075 (7.9)	1270 (11.8)	12,805 (7.6)	< 0.0001
Charlson Comorbidity Index*				< 0.0001
0	21,574 (12.1)	975 (9.1)	20,599 (12.3)	
1	40,912 (22.9)	2014 (18.8)	38,898 (23.2)	
≥ 2	116,100 (65.0)	7743 (72.2)	108,357 (64.6)	
Healthcare visit frequency*^,†^				< 0.0001
First quartile	44,748 (25.1)	1904 (17.7)	42,844 (25.5)	
Second quartile	44,669 (25.0)	2325 (21.7)	42,344 (25.2)	
Third quartile	44,595 (25.0)	2751 (25.6)	41,844 (24.9)	
Fourth quartile	44,574 (25.0)	3752 (35.0)	40,822 (24.3)	
Medication history*				
HMG-CoA reductase inhibitors	41,904 (23.5)	2611 (24.3)	39,293 (23.4)	0.029
Antidiabetic medication	28,439 (15.9)	2056 (19.2)	26,383 (15.7)	< 0.0001
Antihypertensive medication	106,098 (59.4)	6101 (62.4)	99,397 (59.2)	< 0.0001
Antidepressants	10,318 (5.8)	1028 (9.6)	9290 (5.5)	< 0.0001
Benzodiazepine and sleep pill	47,203 (26.4)	3835 (35.7)	43,368 (25.8)	< 0.0001
Antiplatelet medication	50,994 (28.6)	3491 (32.5)	47,503 (28.3)	< 0.0001
Laboratory findings, mean (SD)				
Cholesterol level, mg/dL^‡^				
Total cholesterol	196.4 (38.4)	198.6 (39.3)	196.3 (38.4)	< 0.0001
LDL-C	116.2 (35.3)	117.6 (35.8)	116.1 (35.2)	< 0.0001
HDL-C	52.8 (13.4)	52.9 (13.7)	52.8 (13.4)	0.211
Triglyceride	135.6 (68.6)	138.6 (69.9)	135.4 (68.5)	< 0.0001
Fasting glucose, mg/dL^‡^	101.9 (21.0)	102.8 (22.3)	101.9 (20.9)	< 0.0001
Hemoglobin, g/dL^‡^	13.3 (1.5)	13.0 (1.4)	13.3 (1.5)	< 0.0001
Physical examination findings, mean (SD)^‡^				
Systolic blood pressure, mmHg	130.3 (15.9)	130.4 (16.4)	130.3 (15.9)	0.534
Diastolic blood pressure, mmHg	78.0 (9.8)	78.1 (10.1)	78.1 (9.8)	0.728
Body mass index, kg/m^2^	23.8 (3.1)	23.5 (3.2)	23.8 (3.1)	< 0.0001

*Group comparisons by chi-squared tests

^†^The fourth quartile group had the highest frequency of medical visits

^‡^Group comparisons by *T* tests

### Vascular risk factors and the risk of subsequent AD

**Fig. 2:**
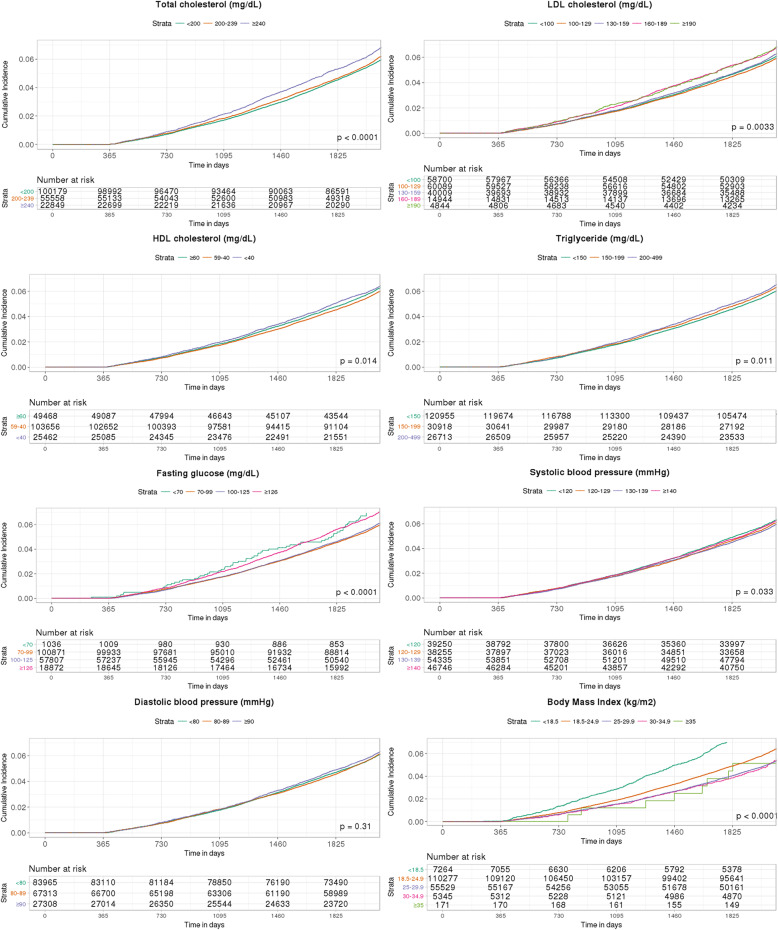
The Kaplan-Meier estimates of incidence of Alzheimer’s disease by vascular risk factors categorized based on the current Korean guidelines among older adults

The cumulative incidence of AD events according to the categorized vascular risk factors based on the current Korean guidelines is presented in Fig. 2. Compared to the referent levels, higher levels of TC, LDL-C, and TG were associated with a higher cumulative incidence of AD, whereas higher levels of BMI showed a lower cumulative AD incidence. Clear linear trends were not observed in other vascular risk factors.

In the age- and sex-adjusted models, highest levels of TC (≥ 240 mg/dL) and TG (200–499 mg/dL) showed a significantly higher risk of AD (TC: HR = 1.085 [95% CI = 1.025–1.149], TG: HR = 1.101 [95% CI = 1.044–1.161]) compared to the referent levels (TC, < 200 mg/dL; TG, < 150 mg/dL), whereas highest SBP levels (≥ 140 mmHg vs. < 120 mmHg) showed a significantly lower AD risk (HR = 0.887 [95% CI = 0.840–0.946]). Other vascular risk factors exhibited no significant associations with the risk of AD (Table 2 and Additional file 1: Table S2).

**Table 2:** Adjusted hazard ratio (aHR) of Alzheimer’s disease according to vascular risk factors

	Group 1	Group 2	Group 3	Group 4	Group 5	***P*** for trend	Sex × group interaction ***P***
**Total cholesterol**							
Ranges (mg/dL)	< 200	200–239	≥ 240	–	–		
Total population, age- and sex-aHR	1 [reference]	1.013 (0.971–1.058)	1.085 (1.025–1.149)	–	–	0.013	
Total population, aHR^†^	1 [reference]	1.035 (0.991–1.082)	1.089 (1.025–1.156)	–	–	0.005	
Male, aHR^†^	1 [reference]	0.998 (0.924–1.079)	1.055 (0.929–1.198)	–	–	0.574	0.339
Female, aHR^†^	1 [reference]	1.055 (1.000–1.114)	1.102 (1.028–1.181)	–	–	0.004	
**LDL cholesterol**							
Ranges (mg/dL)	< 100	100–129	130–159	160–189	≥ 190		
Total population, age- and sex-aHR	1 [reference]	0.952 (0.908–0.997)	0.981 (0.932–1.034)	1.045 (0.973–1.122)	1.017 (0.906–1.142)	0.658	
Total population, aHR^‡^	1 [reference]	1.006 (0.959–1.055)	1.046 (0.991–1.103)	1.116 (1.039–1.199)	1.085 (0.966–1.218)	0.004	
Male, aHR^‡^	1 [reference]	0.957 (0.887–1.033)	0.963 (0.878–1.056)	1.073 (0.932–1.235)	1.123 (0.855–1.474)	0.928	0.208
Female, aHR^‡^	1 [reference]	1.044 (0.981–1.111)	1.096 (1.026–1.171)	1.150 (1.056–1.252)	1.095 (0.961–1.247)	0.0003	
**HDL cholesterol**							
Ranges (mg/dL)	≥ 60	59–40	< 40	–	–		
Total population, age- and sex-aHR	1 [reference]	0.965 (0.924–1.008)	1.000 (0.940–1.062)			0.528	
Total population, aHR^§^	1 [reference]	0.945 (0.904–0.988)	0.941 (0.882–1.004)			0.019	
Male, aHR^§^	1 [reference]	0.953 (0.880–1.031)	0.978 (0.880–1.087)	–	–	0.517	0.132
Female, aHR^§^	1 [reference]	0.943 (0.894–0.995)	0.913 (0.840–0.991)	–	–	0.012	
**Triglyceride**							
Ranges (mg/dL)	< 150	150–199	200–499	–	–		
Total population, age- and sex-aHR	1 [reference]	1.031 (0.981–1.085)	1.101 (1.044–1.161)	–	–	0.0004	
Total population, aHR^||^	1 [reference]	1.030 (0.978–1.084)	1.094 (1.034–1.156)		–	0.002	
Male, aHR^||^	1 [reference]	1.100 (1.003–1.205)	1.084 (0.978–1.201)	–	–	0.044	0.189
Female, aHR^||^	1 [reference]	1.002 (0.941–1.067)	1.100 (1.030–1.176)		–	0.011	
**Fasting glucose**							
Ranges (mg/dL)	< 70	70–99	100–125	≥ 126	–		
Total population, age- and sex-aHR	1 [reference]	0.850 (0.671–1.078)	0.868 (0.684–1.102)	1.029 (0.807–1.311)	–	< .0001	
Total population, aHR^#^	1 [reference]	0.897 (0.707–1.138)	0.882 (0.695–1.120)	0.896 (0.702–1.143)	–	0.689	
Male, aHR^#^	1 [reference]	0.839 (0.577–1.219)	0.825 (0.567–1.200)	0.907 (0.618–1.330)	–	0.358	0.09
Female, aHR^#^	1 [reference]	0.932 (0.685–1.268)	0.918 (0.674–1.250)	0.891 (0.650–1.222)	–	0.27	
**Systolic blood pressure**							
Ranges (mmHg)	< 120	120–129	130–139	≥ 140	–		
Total population, age- and sex-aHR	1 [reference]	0.951 (0.898–1.007)	0.890 (0.844–0.938)	0.887 (0.840–0.936)	–	< .0001	
Total population, aHR**	1 [reference]	0.925 (0.872–0.982)	0.854 (0.805–0.905)	0.827 (0.771–0.888)	–	< .0001	
Male, aHR**	1 [reference]	0.839 (0.577–1.219)	0.825 (0.567–1.200)	0.907 (0.618–1.330)	–	0.358	0.405
Female, aHR**	1 [reference]	0.884 (0.821–0.953)	0.830 (0.772–0.893)	0.800 (0.734–0.873)	–	< .0001	
**Diastolic blood pressure**							
Ranges (mmHg)	< 80	80–89	≥ 90	–	–		
Total population, age- and sex-aHR	1 [reference]	0.988 (0.948–1.030)	0.984 (0.931–1.040)	–	–	0.5	
Total population, aHR^††^	1 [reference]	1.041 (0.996–1.089)	1.110 (1.037–1.188)	–	–	0.003	
Male, aHR^††^	1 [reference]	1.035 (0.960–1.116)	1.018 (0.904–1.147)	–	–	0.565	0.201
Female, aHR^††^	1 [reference]	1.046 (0.990–1.106)	1.158 (1.066–1.259)	–	–	0.001	
**Body mass index**							
Ranges	< 18.5	18.5–24.9	25–29.9	30–34.9	≥ 35		
Total population, age- and sex-aHR	1 [reference]	1.000 (0.918–1.089)	0.958 (0.876–1.049)	0.967 (0.840–1.114)	1.397 (0.769–2.538)	0.145	
Total population, aHR^‡‡^	1 [reference]	0.981 (0.899–1.069)	0.914 (0.832–1.003)	0.901 (0.780–1.040)	1.292 (0.710–2.349)	0.067	
Male, aHR^‡‡^	1 [reference]	1.065 (0.902–1.233)	1.004 (0.854–1.181)	1.117 (0.818–1.525)	2.428 (0.602–9.792)	0.078	0.536
Female, aHR^‡‡^	1 [reference]	0.929 (0.834–1.034)	0.859 (0.765–0.964)	0.828 (0.702–0.978)	1.100 (0.567–2.135)	0.265	

^†^Adjusted for sex, age, income, lifestyles (smoking, alcohol consumption, and exercise), comorbidities, healthcare visit frequency, medication history, body mass index, systolic/diastolic blood pressure, HDL-C, triglyceride, fasting glucose, and hemoglobin. Sex was not included as a covariate in the sex subgroup analysis

^‡^Adjusted for sex, age, income, lifestyles (smoking, alcohol consumption, and exercise), comorbidities, healthcare visit frequency, medication history, body mass index, systolic/diastolic blood pressure, HDL-C, triglyceride, fasting glucose, and hemoglobin. Sex was not included as a covariate in the sex subgroup analysis

^§^Adjusted for sex, age, income, lifestyles (smoking, alcohol consumption, and exercise), comorbidities, healthcare visit frequency, medication history, body mass index, systolic/diastolic blood pressure, LDL-C, triglyceride, fasting glucose, and hemoglobin. Sex was not included as a covariate in the sex subgroup analysis

^||^Adjusted for sex, age, income, lifestyles (smoking, alcohol consumption, and exercise), comorbidities, healthcare visit frequency, medication history, body mass index, systolic/diastolic blood pressure, LDL-C, HDL-C, fasting glucose, and hemoglobin. Sex was not included as a covariate in the sex subgroup analysis

^#^Adjusted for sex, age, income, lifestyles (smoking, alcohol consumption, and exercise), comorbidities, healthcare visit frequency, medication history, body mass index, systolic/diastolic blood pressure, LDL-C, HDL-C, triglyceride, and hemoglobin. Sex was not included as a covariate in the sex subgroup analysis

^**^Adjusted for sex, age, income, lifestyles (smoking, alcohol consumption, and exercise), comorbidities, healthcare visit frequency, medication history, body mass index, diastolic blood pressure, LDL-C, HDL-C, triglyceride, fasting glucose, and hemoglobin. Sex was not included as a covariate in the sex subgroup analysis

^††^Adjusted for sex, age, income, lifestyles (smoking, alcohol consumption, and exercise), comorbidities, healthcare visit frequency, medication history, body mass index, systolic blood pressure, LDL-C, HDL-C, triglyceride, fasting glucose, and hemoglobin. Sex was not included as a covariate in the sex subgroup analysis

^‡‡^Adjusted for sex, age, income, lifestyles (smoking, alcohol consumption, and exercise), comorbidities, healthcare visit frequency, medication history, systolic/diastolic blood pressure, LDL-C, HDL-C, triglyceride, fasting glucose, and hemoglobin. Sex was not included as a covariate in the sex subgroup analysis

**Fig. 3:**
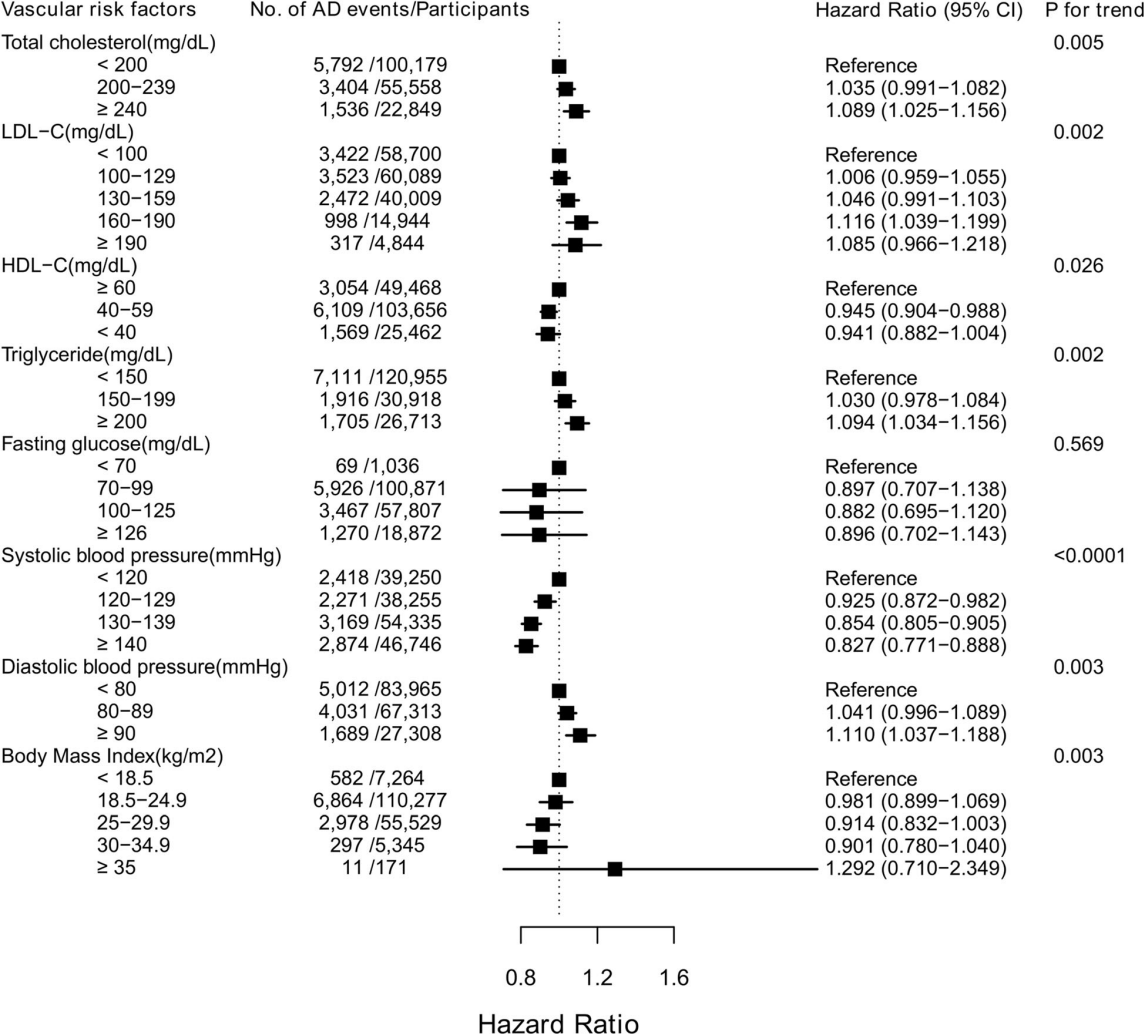
Risk of Alzheimer’s disease in older adults according to vascular risk factors categorized based on the current Korean guidelines. Vascular risk factors were sorted in order of guideline. The subjects with the recommended value (not the lowest value) were used as a reference group

The fully aHRs for AD incidence by vascular risk factors categorized according to the current Korean guidelines are presented in Fig. 3, Table 2, and Additional file 1: Table S2.

The associations of highest levels of TC, TG, and SBP with the incident AD remained significant in the full model (TC: HR = 1.089 [95% CI = 1.025–1.156], TG: HR = 1.094 [95% CI = 1.034–1.156], SBP: HR = 0.827 [95% CI = 0.771–0.888]). The risk of incident AD increased according to LDL-C levels until they reached 190 mg/dL (HR = 1.116 [95% CI = 1.039–1.199] vs. < 100 mg/dL), at which point the risk decreased (HR = 1.085 [95% CI = 0.966–1.218]). In contrast, the risk of incident AD increased according to LDL-C levels until they reached 190 mg/dL (HR = 1.116 [95% CI = 1.039–1.199] vs. < 100 mg/dL), at which point the risk decreased (HR = 1.085 [95% CI = 0.966–1.218]). In contrast, HDL-C levels of 40–59 mg/dL were associated with a significantly lower risk of AD events (HR = 0.945 [95% CI = 0.904–0.988]) than the reference level (≥ 60 mg/dL), while levels < 40 mg/dL did not significantly reduce the risk (HR = 0.941 [95% CI = 0.882–1.004]). The highest DBP levels (≥ 90 mmHg) were associated with an increased risk of incident AD (HR = 1.110 [95% CI = 1.037–1.188]) compared to the referent level (< 80 mmHg). Finally, AD risk decreased according to BMI until reaching 35.0 kg/m^2^ (HR = 0.901 [95% CI = 0.780–1.040]), following which non-significant increases in risk were observed (HR = 1.292 [95% CI = 0.710–2.349]). All vascular risk factors except FG and BMI showed a significant linear trend (*P* for trend = 0.019 to < 0.0001). The substantial differences in the results of the age- and sex-adjusted model and the fully adjusted model could be attributed to the comprehensive adjustment of various confounders (Additional file 1: Table S3).

**Fig. 4:**
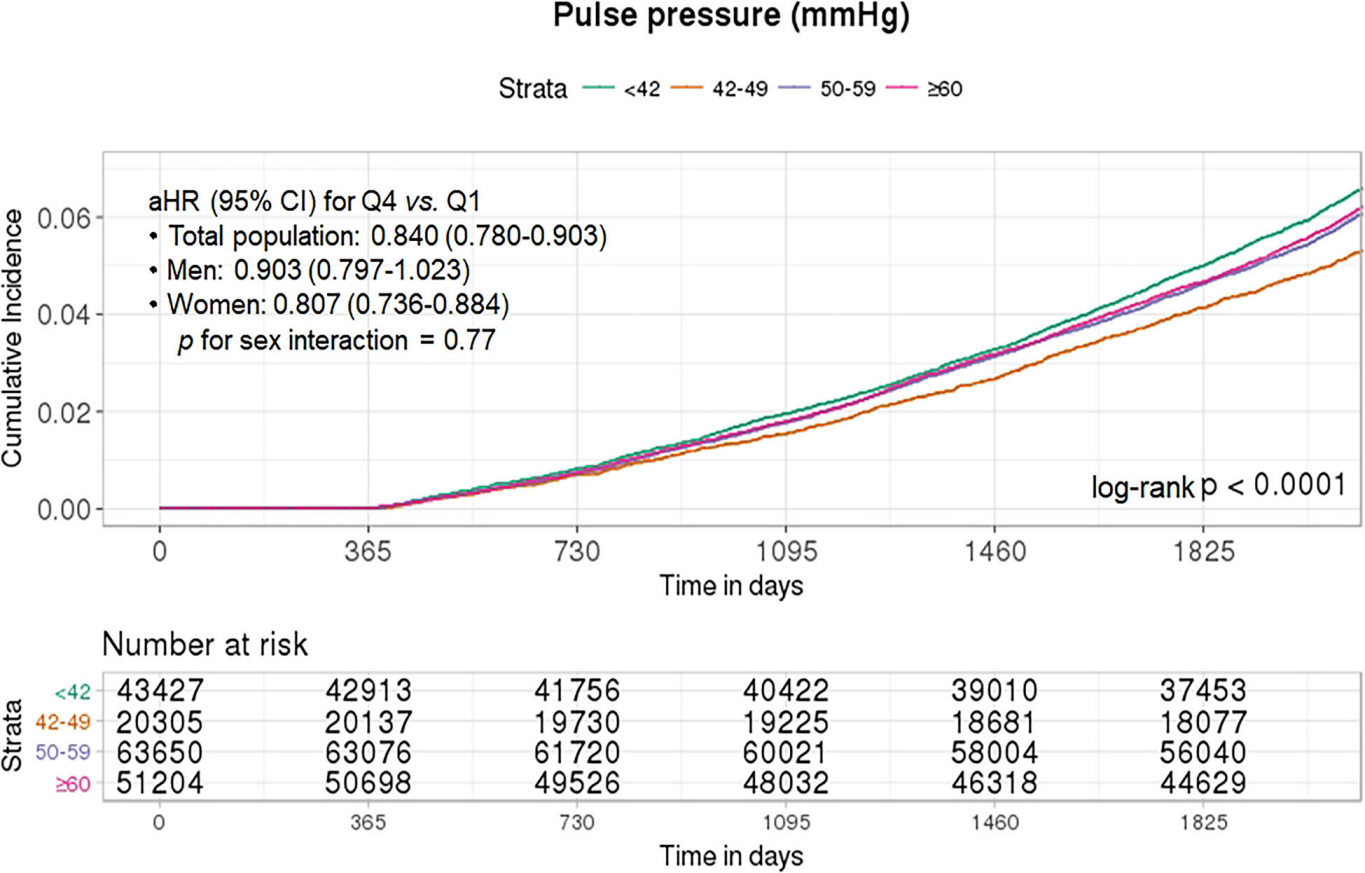
The Kaplan-Meier estimates of incidence of Alzheimer’s disease by pulse pressure categorized based on quartiles among older adults

Figure 4 shows the cumulative incidence of AD events according to the quartile group (Q1–Q4) of PP. Q2–Q4 levels of PP were associated with a lower risk of AD than the Q1 level. In the age- and sex-adjusted model, the Q4 level of PP was significantly associated with a lower risk of AD events (HR for Q4 vs. Q1 = 0.858 [95% CI = 0.815–0.903]). This association remained significant in the fully adjusted model (HR for Q4 vs. Q1 = 0.840 [95% CI = 0.780–0.905]) and exhibited a decreasing linear trend (*P* for trend < 0.0001) (Fig. 4 and Additional file 1: Table S4).

### Subgroup analyses according to sex and medication history

The sex-specific associations between incident AD and vascular risk factors are shown in Table 2 and Additional file 1: Table S5, Table S6. The positive association between AD and the highest TC levels (≥ 240 mg/dL vs. < 200 mg/dL) was significant in women (HR = 1.102 [95% CI = 1.028–1.181]), but not significant in men (HR = 1.055 [95% CI = 0.929–1.198]). In women, higher levels of LDL-C than the referent level (< 100 mg/dL) were associated with a significantly increased risk of AD until they reached 190 mg/dL (HR = 1.150 [95% CI = 1.056–1.252]), at which point the risk decreased (HR = 1.095 [95% CI = 0.961–1.247]), and lower levels of HDL-C than the referent level (≥ 60 mg/dL) were associated with a significantly lower risk of AD (40–59 mg/dL: HR = 0.943 [95% CI = 0.894–0.995], < 40 mg/dL: HR = 0.913 [95% CI = 0.840–0.991], trend *P* = 0.01). In contrast, these associations with LDL-C and HDL-C were not significant in men. The positive association of AD with higher TG levels than the referent level (< 150 mg/dL) was observed in both sex groups. In men, TG levels of 150–199 mg/dL (HR = 1.100 [95% CI = 1.103–1.205]) were significant, but levels ≥ 200 mg/dL (HR = 1.084 [95% CI = 0.978–1.201]) were not significant. In women, only levels ≥ 200 mg/dL exhibited a significant association (HR = 1.100 [95% CI = 1.030–1.176]). Higher DBP (≥ 90 mmHg) was associated with an increased risk of incident AD (HR = 1.158 [95% CI = 1.066–1.259]). However, increasing SBP (up to ≥ 140 mmHg) was linearly associated with decreases in the risk of incident AD (HR = 0.800, [95% CI = 0.734–0.873]) in women, but not significant in men. Higher BMI levels than the referent level (18.5 kg/m^2^) showed a significant negative association with AD risk until reaching 35.0 kg/m^2^ (HR = 0.828 [95% CI = 0.702–0.978]) in women, whereas it showed a positive non-significant association in men (≥ 35.0 kg/m^2^: HR = 2.428 [95% CI = 0.062–9.792]). Compared to Q1 levels, Q4 levels of PP were associated with a lower risk of AD in both sex groups, but it was not significant in men (Fig. 4 and Additional file 1: Table S4). However, these differences were not significant for all vascular risk factors (*P* for interaction = 0.09–0.77).

Furthermore, there was no evidence of an interaction between vascular risk factors and medication during the first 3 years of follow-up regarding incident AD (all *P* for interaction > 0.10, Additional file 1: Table S7).

## Discussion

In this nationwide population-based study of 178,586 Korean older adults, we investigated the association between eight vascular risk factors and the risk of incident AD. As summarized in Fig. 3, lipid profiles including TC, LDL-C, HDL-C, and TG exhibited positive associations with AD risk. SBP and BMI exhibited a negative association with AD incidence, except for the highest group. The risk of AD exhibited a significantly positive association with DBP. Subgroup analyses revealed a significant positive association between TC, LDL-C, HDL-C, TG, DBP, and AD risk in women. Otherwise, a significantly negative association between AD risk and SBP was observed in women. A significant positive association between AD risk and TG was observed in men.

The largest recent meta-analysis, which included 23,338 participants, reported no association between levels of lipid profiles (including TC, LDL-C, and TG) in late-life and AD risk [23]. However, this analysis did not include other potentially confounding vascular risk factors such as BP, FG, or BMI. We observed a positive association between AD risk and lipid profiles including TC, LDL-C, and TG, consistent with recent guidelines [24]. The association between increased cholesterol levels and risk of AD is yet to be fully understood, for the levels in the brain are independent of peripheral tissues due to the blood-brain barrier. However, considering high-fat diets are related to amyloid-beta (Aβ) accumulation, the increased flux of oxysterols to the brain caused by this diet could provide an explanation for our results [25]. Increased influx of oxysterols to the brain is reported to accelerate cognitive deficits in AD [25].

Notably, the direction of the association between HDL-C and AD risk differed from that reported in previous observational studies [26, 27] and guidelines. A recent meta-analysis reported no significant association between HDL-C and AD risk [23]. The protective effects of HDL-C on AD risk have been observed in middle-aged adults, while studies examining patients ≥ 70 years of age have reported no such association [28]. Furthermore, these observational studies had limited sample sizes of < 10,000 [23]. Accumulating evidence suggests that steady-state HDL-C levels are not causally protective against cardiovascular disease [29, 30]. Previous studies have shown that aging alters HDL-C composition, resulting in functional impairment, especially in antioxidant properties [31]. Not HDL quantity but HDL functional quality is thought to impact vascular risks by altered oxidation, inflammation, and thrombosis especially with reverse cholesterol transport [30, 31]. These age-related changes in HDL-C could be related to the different effects of HDL-C on AD according to age group. Due to the age restrictions of the sample, we could not include a middle age comparison population. In addition, our data did not include variables for HDL quality. Further studies including HDL-C composition and function across diverse age groups are warranted.

We observed a conflicting effect of DBP and SBP on AD. We found a negative association between SBP and AD, and a positive association between DBP and AD. These results were also confirmed in analysis for the association between PP and AD (Fig. 4). The association between BP in older adults and cognitive impairment remains uncertain, and the results of observational studies involving older adults are inconsistent [32–34]. Several randomized controlled trials have suggested that lowering BP exerts beneficial effects on cognitive function; however, the evidence is still limited [35–37]. Although the World Health Organization recommends antihypertensive treatment for dementia prevention [24], the American College of Physicians and the American Academy of Family Physicians [38] had previously suggested less strict SBP control for maintaining cognitive health in older adults. Our findings may indicate that this discrepancy is due to the different effects of SBP and DBP on dementia. Inverse associations of SBP with the risk of AD have been reported in large, population-based health studies [39] and recently supported by a Mendelian randomization study of AD [40]. Although SBP cannot fully represent sufficiency of cerebral blood flow, SBP is negatively associated with AD incidence, which could suggest sufficient cerebral oxygen levels follow insufficient cerebral blood flow. Whether this association is derived from cerebral blood flow related to SBP or from the usage of antihypertensive medication needs further neuroimaging research in relation to cerebral blood flow. A previous report on midlife DBP could support our finding, suggesting DBP as the strongest predictor of progression of arterial stiffness, leading to AD [41]. We found a negative association between PP and AD risk, which was inconsistent with previous reports that showed that higher PP in midlife increases AD risk and Aβ transport dysfunction [42]. This discrepancy suggests that the effect of PP on AD risk could differ according to the age group. However, given the observational nature of our study, we could not determine whether there is a causal association between BP and dementia risk.

The association between BMI and AD risk in the present study is consistent with that reported in previous studies [16]. The protective effect of BMI may be related to the role of leptin in regulating hippocampal synaptic plasticity [43] and the additional positive effects of muscle preservation. However, these findings should be interpreted with caution to avoid a potential reverse causation effect (i.e., weight loss in preclinical AD).

Our results regarding the association between FG and AD risk are inconsistent with those of recent clinical trials that tested the effects of controlling serum glucose levels on reducing the risk of AD [44, 45]. However, these trials were conducted in patients with diabetes mellitus or measured restricted outcomes such as an amyloid deposition [44]. The association between FG and AD could differ in the general population.

In the present study, we observed differences in the association between lipid profiles and AD risk according to sex. TC and LDL-C were independent risk factors for AD in the female subgroup, whereas TG was a risk factor in both males and females. The incidence, risk factors, clinical symptoms, and progression of AD may differ between men and women [46–48]. Such sex-based differences may be due to the differences in lipid metabolism and hormone levels. For example, estrogen, which affects cholesterol removal from the liver, plays a significant role in sex-specific morbidity related to vascular risk factors [49]. These distinctive differences may also be driven by different apolipoprotein profiles in men and women. Further studies are required to determine the mechanisms underlying such sex-based differences.

The effect size for associations between vascular risk factors and AD risk was small in the present study (Fig. 3). Moreover, medication use did not modify this association (Additional file 1: Table S7). These findings suggest that management of vascular risk factors alone would not be enough to reduce AD risk. An integrative approach including management of vascular risk factors, lifestyle modifications and cognitive training could have drawn contribution to AD prevention in older adults [50]. However, it should be noted that the effect of controlling vascular risk factors may be stronger in early AD stages or mild cognitive disorders. Once the pathological change has progressed enough to require medication, prevention efforts could not reach significant effects. This could explain the small effect size of vascular risk factors on AD and the little significance of medication modification in our results.

Our large-scale study is advantageous in that we utilized nationwide real-world data representing older adults in the Korean population. Moreover, we addressed multiple vascular risk factors and stratified analyses according to sex and age groups. As most other studies have focused on midlife vascular risk factors related to the risk of dementia, our findings may provide further explanations, representing a clear association with those related to the risk in older adults. This study also had several limitations. First, although we comprehensively adjusted for various confounders including socioeconomic or lifestyle factors, we did not consider potential confounders such as education, occupational attainment, exercise, diet, sufficient sleep, family history, and genetics (apolipoprotein E genotype). The lack of information on the level of hemoglobin A1c, a standard tool to determine average blood glucose control levels, could be a significant limitation, which needs to be elaborated through future research representing actual glucose control. Second, the operational definition of AD may be associated with misdiagnosis or underdiagnosis. However, the rate of AD in our study population was similar to rates reported in epidemiological studies conducted in South Korea [51]. Additionally, we included prescription information in the operational criteria to improve the accuracy of diagnosis. In South Korea, clinicians are required to document clinical diagnoses of dementia as well as the results of neuropsychological tests in order to prescribe the medications outlined in our inclusion criteria. Dementia ascertainment, defined as prescribing cognitive enhancer, could have limited information to fully represent the actual onset of AD, considering the insidious progress of AD in the clinical situation. Lack of information regarding biomarkers and AD confirmation via imaging might have led to the limitation of a heterogeneous group with numerous contributing pathological substrates. The lack of information related to the severity stage of AD also had limitation to observe association between the role of vascular risk factors and the course of AD. Third, because the study population included individuals from a single country, our findings may not be generalizable to people from other countries. Fourth, given the observational nature of our study, we could not determine causal relationships between vascular risk factors and AD risk. And careful interpretation should be conducted with respect to reverse causation effect, for changes in vascular metrics occur proximal to the onset of major neurocognitive dysfunction rather than early stages of AD pathophysiology. Last, since our study population was based on the National Health Insurance Service-Senior Cohort (≥ 60 years), we could not investigate whether midlife and later life vascular risk factors have different effects on incident AD. Future studies examining the differences among midlife and later life vascular risk factors from a same population would be informative.

## Conclusions

In the present study, we observed a positive association between AD risk and TC, LDL-C, HDL-C, TG, and DBP among older adults. Further, SBP, BMI, and PP exhibited robust negative associations with AD risk. Associations between AD and TC, LDL-C, HDL-C, and DBP differed according to sex. More elaborate, prospective studies are required to gain a greater understanding of the association between vascular risk factors and AD in older adults.

## Supplementary information

**Additional file 1: Table S1.** The current Korean guidelines for vascular risk factors. **Table S2.** Detailed results for risk of Alzheimer’s disease according to vascular risk factors. **Table S3.** Adjusted hazard ratios of risk factors associated with incident Alzheimer’s disease by Cox proportional hazards model (*n*= 178,586). **Table S4.** Risk of Alzheimer’s disease in older adults according to pulse pressure categorized based on quartiles. **Table S5.** Risk of Alzheimer’s disease according to vascular risk factors in male subgroup (*n*=81,242). **Table S6.** Risk of Alzheimer’s disease according to vascular risk factors in female subgroup (*n*=97,344). **Table S7.** Stratified analysis according to medications prescription during the first 3 years of follow-up.

## Data Availability

This study is based on National Health Insurance Service (NHIS) registry data in South Korea (NHIS-2018-2-198). Because these data belong to the NHIS, the authors are not permitted to share them, except in aggregate (as, for example, in a publication). However, interested parties can obtain the data on which the study was based by submitting a research protocol to the NHIS (https://nhiss.nhis.or.kr/bd/ab/bdaba000eng.do). The analytic/statistical codes are available from the corresponding author (wjmyung@snubh.org, WM), upon reasonable request.

## Abbreviations

AD: Alzheimer’s disease
aHR: Adjusted hazard ratio
Aβ: Amyloid-beta
BP: Blood pressure
BMI: Body mass index
DALY: Disability-adjusted life year
DBP: Diastolic blood pressure
FG: Fasting glucose
HDL-C: High-density lipoprotein cholesterol
ICD-10: International Classification of Diseases, Tenth Revision
LDL-C: Low-density lipoprotein cholesterol
NHID: National Health Insurance Database
NHIS-SC: National Health Insurance Service-Senior Cohort
PH: Proportional hazard
PP: Pulse pressure
SBP: Systolic blood pressure
TC: Total cholesterol
TG: Triglycerides
VIF: Variance inflation factor
