# Supplementary material for: Associations between vascular risk factors and subsequent Alzheimer’s disease in older adults
Source: Alzheimers Res Ther. 2020 Sep 26;12:117. doi: 10.1186/s13195-020-00690-7 (PMC7520023; doi:10.1186/s13195-020-00690-7)
Supplement: Supplementary file 1 — Additional file 1: Table S1. The current Korean guidelines for vascular risk factors. Table S2. Detailed results for risk of Alzheimer’s disease according to vascular risk factors. Table S3. Adjusted hazard ratios of risk factors associated with incident Alzheimer’s disease by Cox proportional hazards model (n= 178,586). Table S4. Risk of Alzheimer’s disease in older adults according to pulse pressure categorized based on quartiles. Table S5. Risk of Alzheimer’s disease according to vascular risk factors in male subgroup (n=81,242). Table S6. Risk of Alzheimer’s disease according to vascular risk factors in female subgroup (n=97,344). Table S7. Stratified analysis according to medications prescription during the first 3 years of follow-up. [file 13195_2020_690_MOESM1_ESM.doc]

**Associations between vascular risk factors and subsequent Alzheimer’s disease in older adults**

**Supplemental Online Material**

**Table S1. The current Korean guidelines for vascular risk factors.**

|  | **Group 1** | **Group 2** | **Group 3** | **Group 4** | **Group 5** |
| --- | --- | --- | --- | --- | --- |
| **Total cholesterol (mg/dL)** | < 200 | 200 – 239 | ≥ 240 |  |  |
| **LDL cholesterol (mg/dL)** | < 100 | 100 – 129 | 130 – 159 | 160 – 189 | ≥ 190 |
| **HDL cholesterol (mg/dL)** | ≥ 60 | 59 – 40 | < 40 |  |  |
| **Triglyceride (mg/dL)** | < 150 | 150 – 199 | 200 – 499 |  |  |
| **Fasting glucose (mg/dL)** | < 70 | 70 – 99 | 100 – 125 | ≥ 126 |  |
| **Systolic blood pressure (mmHg)** | < 120 | 120 – 129 | 130 – 139 | ≥ 140 |  |
| **Diastolic blood pressure (mmHg)** | < 80 | 80 – 89 | ≥ 90 |  |  |
| **Body Mass Index (kg/m^2^)** | < 18.5 | 18.5 – 24.9 | 25 – 29.9 | 30 – 34.9 | ≥ 35 |

**Table S2. Detailed results for risk of Alzheimer’s disease according to vascular risk factors.**

|  | **Group 1** | **Group 2** | **Group 3** | **Group 4** | **Group 5** | **P for trend** |
| --- | --- | --- | --- | --- | --- | --- |
| **Total cholesterol** |  |  |  |  |  |  |
| Ranges (mg/dL) | < 200 | 200 – 239 | ≥ 240 | - | - |  |
| Alzheimer’s disease events | 5792 | 3404 | 1536 |  |  |  |
| Person-years | 556805.8 | 313387.5 | 128938.0 |  |  |  |
| Incidence ^a^. | 1040.2 | 1086.2 | 1191.3 |  |  |  |
| Age- and sex-adjusted hazard ratios | 1 [Reference] | 1.013 (0.971-1.058) | 1.085 (1.025-1.149) |  |  | 0.013 |
| Adjusted hazard ratios ^b^. | 1 [Reference] | 1.035 (0.991-1.082) | 1.089 (1.025-1.156) |  |  | 0.005 |
| **LDL-C** |  |  |  |  |  |  |
| Ranges (mg/dL) | < 100 | 100 – 129 | 130 – 159 | 160 – 189 | ≥ 190 |  |
| Alzheimer’s disease events | 3422 | 3523 | 2472 | 998 | 317 |  |
| Person-years | 324812.6 | 337335.5 | 225605.7 | 84258.4 | 27119.2 |  |
| Incidence ^a^. | 1053.5 | 1044.4 | 1095.7 | 1184.5 | 1168.9 |  |
| Age- and sex-adjusted hazard ratios | 1 [Reference] | 0.952 (0.908-0.997) | 0.981 (0.932-1.034) | 1.045 (0.973-1.122) | 1.017 (0.906-1.142) | 0.658 |
| Adjusted hazard ratios ^c^. | 1 [Reference] | 1.006 (0.959-1.055) | 1.046 (0.991-1.103) | 1.116 (1.039-1.199) | 1.085 (0.966-1.218) | 0.004 |
| **HDL-C** |  |  |  |  |  |  |
| Ranges (mg/dL) | ≥ 60 | 59 – 40 | < 40 | - | - |  |
| Alzheimer’s disease events | 3054 | 6109 | 1569 |  |  |  |
| Person-years | 277862.2 | 581380.1 | 139889.1 |  |  |  |
| Incidence ^a^. | 1099.1 | 1050.8 | 1121.6 |  |  |  |
| Age- and sex-adjusted hazard ratios | 1 [Reference] | 0.965 (0.924-1.008) | 1.000 (0.940-1.062) |  |  | 0.528 |
| Adjusted hazard ratios ^d^. | 1 [Reference] | 0.945 (0.904-0.988) | 0.941 (0.882-1.004) |  |  | 0.019 |
| **Triglyceride** |  |  |  |  |  |  |
| Ranges (mg/dL) | < 150 | 150 – 199 | 200 – 499 | - | - |  |
| Alzheimer’s disease events | 7111 | 1916 | 1705 |  |  |  |
| Person-years | 675359.3 | 173601.23 | 150170.8 |  |  |  |
| Incidence ^a^. | 1052.9 | 1103.7 | 1135.4 |  |  |  |
| Age- and sex-adjusted hazard ratios | 1 [Reference] | 1.031 (0.981-1.085) | 1.101 (1.044-1.161) |  |  | 0.0004 |
| Adjusted hazard ratios ^e^. | 1 [Reference] | 1.030 (0.978-1.084) | 1.094 (1.034-1.156) |  |  | 0.002 |
| **Fasting glucose** |  |  |  |  |  |  |
| Ranges (mg/dL) | < 70 | 70 – 99 | 100 – 125 | ≥ 126 |  |  |
| Alzheimer’s disease events | 69 | 5926 | 3467 | 1270 |  |  |
| Person-years | 5576.9 | 566184.1 | 323379.7 | 103990.4 |  |  |
| Incidence ^a^. | 1237.2 | 1046.7 | 1072.1 | 1221.3 |  |  |
| Age- and sex-adjusted hazard ratios | 1 [Reference] | 0.850 (0.671-1.078) | 0.868 (0.684-1.102) | 1.029 (0.807-1.311) |  | <.0001 |
| Adjusted hazard ratios ^f^. | 1 [Reference] | 0.897 (0.707-1.138) | 0.882 (0.695-1.120) | 0.896 (0.702-1.143) |  | 0.689 |
| **Systolic blood pressure** |  |  |  |  |  |  |
| Ranges (mmHg) | < 120 | 120 – 129 | 130 – 139 | ≥ 140 |  |  |
| Alzheimer’s disease events | 2418 | 2271 | 3169 | 2874 |  |  |
| Person-years | 218362.5 | 214642.5 | 305006.6 | 261119.8 |  |  |
| Incidence ^a^. | 1107.3 | 1058.0 | 1039.0 | 1100.6 |  |  |
| Age- and sex-adjusted hazard ratios | 1 [Reference] | 0.951 (0.898-1.007) | 0.890 (0.844-0.938) | 0.887 (0.840-0.936) |  | <.0001 |
| Adjusted hazard ratios ^g^. | 1 [Reference] | 0.925 (0.872-0.982) | 0.854 (0.805-0.905) | 0.827 (0.771-0.888) |  | <.0001 |
| **Diastolic blood pressure** |  |  |  |  |  |  |
| Ranges (mmHg) | < 80 | 80 – 89 | ≥ 90 | - |  |  |
| Alzheimer’s disease events | 5012 | 4031 | 1689 |  |  |  |
| Person-years | 469821.0 | 377109.8 | 152200.6 |  |  |  |
| Incidence | 1066.8 | 1068.9 | 1109.7 |  |  |  |
| Age- and sex-adjusted hazard ratios | 1 [Reference] | 0.988 (0.948-1.030) | 0.984 (0.931-1.040) |  |  | 0.5 |
| Adjusted hazard ratios ^h^. | 1 [Reference] | 1.041 (0.996-1.089) | 1.110 (1.037-1.188) |  |  | 0.003 |
| **Body Mass Index** |  |  |  |  |  |  |
| Ranges (kg/m^2^) | < 18.5 | 18.5 – 24.9 | 25 – 29.9 | 30 – 34.9 | ≥ 35 |  |
| Alzheimer’s disease events | 582 | 6864 | 2978 | 297 | 11 |  |
| Person-years | 37145.4 | 614334.0 | 316169.9 | 30519.9 | 962.2 |  |
| Incidence | 1566.8 | 1117.3 | 941.9 | 973.1 | 1143.2 |  |
| Age- and sex-adjusted hazard ratios | 1 [Reference] | 1.000 (0.918-1.089) | 0.958 (0.876-1.049) | 0.967 (0.840-1.114) | 1.397 (0.769-2.538) | 0.145 |
| Adjusted hazard ratios ^h^. | 1 [Reference] | 0.981 (0.899-1.069) | 0.914 (0.832-1.003) | 0.901 (0.780-1.040) | 1.292 (0.710-2.349) | 0.067 |

Abbreviations: LDL-C, low-density lipoprotein cholesterol; HDL-C, high-density lipoprotein cholesterol

^a^. incidence was defined as events/100,000 person-years

^b^. Adjusted for sex, age, income, life styles (smoking, alcohol consumption and exercise), co-morbidities, health care visit frequency, medication history, body mass index, systolic/diastolic blood pressure, HDL-C, triglyceride, fasting glucose, and hemoglobin.

^c^. Adjusted for sex, age, income, life styles (smoking, alcohol consumption and exercise), co-morbidities, health care visit frequency, medication history, body mass index, systolic/diastolic blood pressure, HDL-C, triglyceride, fasting glucose, and hemoglobin.

^d^. Adjusted for sex, age, income, life styles (smoking, alcohol consumption and exercise), co-morbidities, health care visit frequency, medication history, body mass index, systolic/diastolic blood pressure, LDL-C, triglyceride, fasting glucose, and hemoglobin.

^e^. Adjusted for sex, age, income, life styles (smoking, alcohol consumption and exercise), co-morbidities, health care visit frequency, medication history, body mass index, systolic/diastolic blood pressure, LDL-C, HDL-C, fasting glucose, and hemoglobin.

^f^. Adjusted for sex, age, income, life styles (smoking, alcohol consumption and exercise), co-morbidities, health care visit frequency, medication history, body mass index, systolic/diastolic blood pressure, LDL-C, HDL-C, triglyceride, and hemoglobin.

^g^. Adjusted for sex, age, income, life styles (smoking, alcohol consumption and exercise), co-morbidities, health care visit frequency, medication history, body mass index, diastolic blood pressure, LDL-C, HDL-C, triglyceride, fasting glucose, and hemoglobin.

^h^. Adjusted for sex, age, income, life styles (smoking, alcohol consumption and exercise), co-morbidities, health care visit frequency, medication history, body mass index, systolic blood pressure, LDL-C, HDL-C, triglyceride, fasting glucose, and hemoglobin.

^i^. Adjusted for sex, age, income, life styles (smoking, alcohol consumption and exercise), co-morbidities, health care visit frequency, medication history, systolic/diastolic blood pressure, LDL-C, HDL-C, triglyceride, fasting glucose, and hemoglobin.

**Table S3. Adjusted hazard ratios of risk factors associated with incident Alzheimer’s disease by Cox proportional hazards model (n = 178,586).**

|  | Adjusted Hazard Ratio (95% CI) |
| --- | --- |
| Sex |  |
| Male | 1 [Reference] |
| Female | 1.352 (1.279-1.429) |
| Age | 1.160 (1.156-1.163) |
| Income |  |
| Medical Aid | 1 [ Reference ] |
| Low (1^st^–3^rd^ decile) | 0.493 (0.433-0.562) |
| Middle (4^th^–7^th^ decile) | 0.549 (0.483-0.624) |
| High (8^th^–10^th^ decile) | 0.567 (0.500-0.642) |
| Life styles |  |
| Smoking status |  |
| Never smoking | 1 [ Reference ] |
| Ex-smoking | 0.960 (0.896-1.029) |
| Current smoking | 1.089 (1.014-1.170) |
| Alcohol consumption |  |
| Rarely (0–2 days/week) | 1 [ Reference ] |
| Light (3–4 days/week) | 1.014 (0.911-1.128) |
| Heavy (5–7 days/week) | 1.110 (1.014-1.216) |
| Exercise |  |
| No exercise | 1 [ Reference ] |
| Exercise | 0.996 (0.954-1.040) |
| Co-morbidities |  |
| Depression | 1.282 (1.205-1.364) |
| Charlson co-morbidity Index | 1.075 (1.063-1.087) |
| Health care visit frequency |  |
| First quartile | 1 [ Reference ] |
| Second quartile | 1.117 (1.049-1.189) |
| Third quartile | 1.198 (1.123-1.277) |
| Fourth quartile | 1.356 (1.266-1.453) |
| Medication history |  |
| HMG-CoA reductase inhibitors | 0.981 (0.934-1.031) |
| Diabetes medication | 1.156 (1.088-1.227) |
| Antihypertensive drug | 0.874 (0.834-0.916) |
| Antidepressants | 1.309 (1.222-1.402) |
| Benzodiazepine and sleep pill | 1.119 (1.067-1.173) |
| Antiplatelet (Aspirin or Clopidogrel) | 1.063 (1.015-1.114) |
| Laboratory findings |  |
| Cholesterol level |  |
| Low-density lipoprotein cholesterol | 1.001 (1.000-1.001) |
| High-density lipoprotein cholesterol | 1.002 (1.001-1.004) |
| Triglyceride | 1.001 (1.000-1.001) |
| Fasting glucose | 1.000 (0.999-1.001) |
| Hemoglobin | 0.998 (0.983-1.013) |
| Physical examination findings |  |
| Systolic blood pressure | 0.996 (0.994-0.997) |
| Diastolic blood pressure | 1.006 (1.004-1.009) |
| Body Mass Index | 0.989 (0.983-0.996) |

**Table S4. Risk of Alzheimer’s disease in older adults according to pulse pressure categorized based on quartiles.**

|  | Q1 | Q2 | Q3 | Q4 | **P for trend** | **P for interaction** |
| --- | --- | --- | --- | --- | --- | --- |
| **Pulse pressure** |  |  |  |  |  |  |
| Ranges (mmHg) | < 42 | 42 – 49 | 50 – 59 | ≥ 60 |  |  |
| Total population, age- sex- aHR | 1 [Reference] | 0.932 (0.869-1.001) | 0.914 (0.870-0.960) | 0.858 (0.815-0.903) | <.0001 |  |
| Total population, aHR ^i^. | 1 [Reference] | 0.928 (0.864-0.997) | 0.904 (0.855-0.956) | 0.840 (0.780-0.905) | <.0001 |  |
| Male, aHR ^b^. | 1 [Reference] | 0.956 (0.850-1.076) | 0.930 (0.846-1.023) | 0.903 (0.797-1.023) | 0.148 | 0.774 |
| Female, aHR ^b^. | 1 [Reference] | 0.912 (0.833-0.998) | 0.889 (0.830-0.953) | 0.807 (0.736-0.884) | <.0001 |  |

Adjusted for sex, age, income, life styles (smoking, alcohol consumption and exercise), co-morbidities, health care visit frequency, medication history, body mass index, systolic blood pressure, HDL-C, triglyceride, fasting glucose, and hemoglobin.

**Table S5. Risk of Alzheimer’s disease according to vascular risk factors in male subgroup (n=81,242).**

|  | **Group 1** | **Group 2** | **Group 3** | **Group 4** | **Group 5** | **P for trend** |
| --- | --- | --- | --- | --- | --- | --- |
| **Total cholesterol** |  |  |  |  |  |  |
| Ranges (mg/dL) | < 200 | 200 – 239 | ≥ 240 | - | - |  |
| Alzheimer’s disease events | 2509 | 942 | 291 |  |  |  |
| Person-years | 292937.0 | 119555.2 | 35249.7 |  |  |  |
| Incidence ^a^. | 856.5 | 787.9 | 825.5 |  |  |  |
| Age-adjusted hazard ratios | 1 [Reference] | 0.977 (0.907-1.054) | 1.061 (0.940-1.198) | - | - | 0.741 |
| Adjusted hazard ratios ^b^. | 1 [Reference] | 0.998 (0.924-1.079) | 1.055 (0.929-1.198) | - | - | 0.574 |
| **LDL-C** |  |  |  |  |  |  |
| Ranges (mg/dL) | < 100 | 100 – 129 | 130 – 159 | 160 – 190 | ≥ 190 |  |
| Alzheimer’s disease events | 1549 | 1245 | 668 | 226 | 54 |  |
| Person-years | 175185.4 | 155370.3 | 85391.7 | 25570.1 | 6224.5 |  |
| Incidence ^a^. | 884.2 | 801.3 | 782.3 | 883.8 | 867.5 |  |
| Age-adjusted hazard ratios | 1 [Reference] | 0.909 (0.843-0.979) | 0.904 (0.825-0.989) | 1.026 (0.892-1.179) | 1.056 (0.805-1.385) | 0.125 |
| Adjusted hazard ratios ^c^. | 1 [Reference] | 0.957 (0.887-1.033) | 0.963 (0.878-1.056) | 1.073 (0.932-1.235) | 1.123 (0.855-1.474) | 0.928 |
| **HDL-C** |  |  |  |  |  |  |
| Ranges (mg/dL) | ≥ 60 | 59 – 40 | < 40 | - | - |  |
| Alzheimer’s disease events | 934 | 2120 | 688 |  |  |  |
| Person-years | 112256.7 | 260694.4 | 74790.8 |  |  |  |
| Incidence ^a^. | 832.0 | 813.2 | 919.9 |  |  |  |
| Age-adjusted hazard ratios | 1 [Reference] | 0.983 (0.910-1.062) | 1.084 (0.982-1.196) | - | - | 0.225 |
| Adjusted hazard ratios ^d^. | 1 [Reference] | 0.953 (0.880-1.031) | 0.978 (0.880-1.087) | - | - | 0.517 |
| **Triglyceride** |  |  |  |  |  |  |
| Ranges (mg/dL) | < 150 | 150 – 199 | 200 – 499 | - | - |  |
| Alzheimer’s disease events | 2676 | 590 | 476 |  |  |  |
| Person-years | 315976.3 | 70017.1 | 61748.6 |  |  |  |
| Incidence ^a^. | 846.9 | 842.7 | 770.9 |  |  |  |
| Age-adjusted hazard ratios | 1 [Reference] | 1.111 (1.016-1.215) | 1.096 (0.994-1.208) | - | - | 0.015 |
| Adjusted hazard ratios ^e^. | 1 [Reference] | 1.100 (1.003-1.205) | 1.084 (0.978-1.201) |  | - | 0.044 |
| **Fasting glucose** |  |  |  |  |  |  |
| Ranges (mg/dL) | < 70 | 70 – 99 | 100 – 125 | ≥ 126 | - |  |
| Alzheimer’s disease events | 28 | 1980 | 1241 | 493 |  |  |
| Person-years | 2613.3 | 242851.9 | 151759.0 | 50517.8 |  |  |
| Incidence ^a^. | 1071.5 | 815.3 | 817.7 | 975.9 |  |  |
| Age-adjusted hazard ratios | 1 [Reference] | 0.788 (0.543-1.144) | 0.802 (0.551-1.166) | 1.038 (0.709-1.520) | - | <.0001 |
| Adjusted hazard ratios ^f^. | 1 [Reference] | 0.839 (0.577-1.219) | 0.825 (0.567-1.200) | 0.907 (0.618-1.330) | - | 0.358 |
| **Systolic blood pressure** |  |  |  |  |  |  |
| Ranges (mmHg) | < 120 | 120 – 129 | 130 – 139 | ≥ 140 | - |  |
| Alzheimer’s disease events | 862 | 839 | 1105 | 936 |  |  |
| Person-years | 99474.3 | 97281.1 | 137102.8 | 113883.7 |  |  |
| Incidence ^a^. | 866.6 | 862.4 | 806.0 | 821.9 |  |  |
| Age-adjusted hazard ratios | 1 [Reference] | 0.999 (0.908-1.099) | 0.896 (0.820-0.980) | 0.884 (0.806-0.970) | - | 0.002 |
| Adjusted hazard ratios ^g^. | 1 [Reference] | 1.002 (0.908-1.106) | 0.899 (0.813-0.993) | 0.879 (0.780-0.990) | - | 0.014 |
| **Diastolic blood pressure** |  |  |  |  |  |  |
| Ranges (mmHg) | < 80 | 80 – 89 | ≥ 90 | - | - |  |
| Alzheimer’s disease events | 1828 | 1390 | 524 |  |  |  |
| Person-years | 211481.8 | 168855.2 | 67404.9 |  |  |  |
| Incidence | 864.4 | 823.2 | 777.4 |  |  |  |
| Age-adjusted hazard ratios | 1 [Reference] | 0.968 (0.903-1.038) | 0.901 (0.817-0.993) | - | - | 0.039 |
| Adjusted hazard ratios ^h^. | 1 [Reference] | 1.035 (0.960-1.116) | 1.018 (0.904-1.147) | - | - | 0.565 |
| **Body Mass Index** |  |  |  |  |  |  |
| Ranges (kg/m^2^) | < 18.5 | 18.5 – 24.9 | 25 – 29.9 | 30 – 34.9 | < 35 |  |
| Alzheimer’s disease events | 204 | 2613 | 871 | 52 | 2 |  |
| Person-years | 18990.0 | 298112.3 | 123992.3 | 6518.9 | 128.4 |  |
| Incidence | 1074.3 | 876.5 | 702.5 | 797.7 | 1557.3 |  |
| Age-adjusted hazard ratios | 1 [Reference] | 1.086 (0.941-1.253) | 1.044 (0.895-1.218) | 1.202 (0.886-1.632) | 2.606 (0.647-10.496) | 0.115 |
| Adjusted hazard ratios ^h^. | 1 [Reference] | 1.065 (0.920-1.233) | 1.004 (0.854-1.181) | 1.117 (0.818-1.525) | 2.428 (0.602-9.792) | 0.078 |

Abbreviations: LDL-C, low-density lipoprotein cholesterol; HDL-C, high-density lipoprotein cholesterol

^a^. incidence was defined as events/100,000 person-years

^b^. Adjusted for sex, age, income, life styles (smoking, alcohol consumption and exercise), co-morbidities, health care visit frequency, medication history, body mass index, systolic/diastolic blood pressure, HDL-C, triglyceride, fasting glucose, and hemoglobin.

^c^. Adjusted for sex, age, income, life styles (smoking, alcohol consumption and exercise), co-morbidities, health care visit frequency, medication history, body mass index, systolic/diastolic blood pressure, HDL-C, triglyceride, fasting glucose, and hemoglobin.

^d^. Adjusted for sex, age, income, life styles (smoking, alcohol consumption and exercise), co-morbidities, health care visit frequency, medication history, body mass index, systolic/diastolic blood pressure, LDL-C, triglyceride, fasting glucose, and hemoglobin.

^e^. Adjusted for sex, age, income, life styles (smoking, alcohol consumption and exercise), co-morbidities, health care visit frequency, medication history, body mass index, systolic/diastolic blood pressure, LDL-C, HDL-C, fasting glucose, and hemoglobin.

^f^. Adjusted for sex, age, income, life styles (smoking, alcohol consumption and exercise), co-morbidities, health care visit frequency, medication history, body mass index, systolic/diastolic blood pressure, LDL-C, HDL-C, triglyceride, and hemoglobin.

^g^. Adjusted for sex, age, income, life styles (smoking, alcohol consumption and exercise), co-morbidities, health care visit frequency, medication history, body mass index, diastolic blood pressure, LDL-C, HDL-C, triglyceride, fasting glucose, and hemoglobin.

^h^. Adjusted for sex, age, income, life styles (smoking, alcohol consumption and exercise), co-morbidities, health care visit frequency, medication history, body mass index, systolic blood pressure, LDL-C, HDL-C, triglyceride, fasting glucose, and hemoglobin.

^i^. Adjusted for sex, age, income, life styles (smoking, alcohol consumption and exercise), co-morbidities, health care visit frequency, medication history, systolic/diastolic blood pressure, LDL-C, HDL-C, triglyceride, fasting glucose, and hemoglobin.

**Table S6. Risk of Alzheimer’s disease according to vascular risk factors in female subgroup (n=97,344).**

|  | **Group 1** | **Group 2** | **Group 3** | **Group 4** | **Group 5** | **P for trend** |
| --- | --- | --- | --- | --- | --- | --- |
| **Total cholesterol** |  |  |  |  |  |  |
| Ranges (mg/dL) | < 200 | 200 – 239 | ≥ 240 | - | - |  |
| Alzheimer’s disease events | 3283 | 2462 | 1245 |  |  |  |
| Person-years | 263868.8 | 193832.3 | 93688.3 |  |  |  |
| Incidence ^a^. | 1244.2 | 1270.2 | 1328.9 |  |  |  |
| Age-adjusted hazard ratios | 1 [Reference] | 1.034 (0.981-1.089) | 1.099 (1.029-1.173) | - | - | 0.006 |
| Adjusted hazard ratios ^b^. | 1 [Reference] | 1.055 (1.000-1.114) | 1.102 (1.028-1.181) | - | - | 0.004 |
| **LDL-C** |  |  |  |  |  |  |
| Ranges (mg/dL) | < 100 | 100 – 129 | 130 – 159 | 160 – 190 | ≥ 190 |  |
| Alzheimer’s disease events | 1873 | 2278 | 1804 | 772 | 263 |  |
| Person-years | 149627.2 | 181965.2 | 140214.0 | 58688.4 | 20894.6 |  |
| Incidence ^a^. | 1251.8 | 1251.9 | 1286.6 | 1315.4 | 1258.7 |  |
| Age-adjusted hazard ratios | 1 [Reference] | 0.985 (0.927-1.047) | 1.027 (0.963-1.096) | 1.067 (0.981-1.160) | 1.029 (0.905-1.171) | 0.107 |
| Adjusted hazard ratios ^c^. | 1 [Reference] | 1.044 (0.981-1.111) | 1.096 (1.026-1.171) | 1.150 (1.056-1.252) | 1.095 (0.961-1.247) | 0.0003 |
| **HDL-C** |  |  |  |  |  |  |
| Ranges (mg/dL) | ≥ 60 | 59 – 40 | < 40 | - | - |  |
| Alzheimer’s disease events | 2120 | 3989 | 881 |  |  |  |
| Person-years | 165605.5 | 320685.7 | 65098.3 |  |  |  |
| Incidence ^a^. | 1280.2 | 1243.9 | 1353.3 |  |  |  |
| Age-adjusted hazard ratios | 1 [Reference] | 0.958 (0.909-1.010) | 0.951 (0.879-1.029) | - | - | 0.108 |
| Adjusted hazard ratios ^d^. | 1 [Reference] | 0.943 (0.894-0.995) | 0.913 (0.840-0.991) | - | - | 0.012 |
| **Triglyceride** |  |  |  |  |  |  |
| Ranges (mg/dL) | < 150 | 150 – 199 | 200 – 499 | - | - |  |
| Alzheimer’s disease events | 4435 | 1326 | 1229 |  |  |  |
| Person-years | 359383.0 | 103584.1 | 88422.3 |  |  |  |
| Incidence ^a^. | 1234.1 | 1280.1 | 1389.9 |  |  |  |
| Age-adjusted hazard ratios | 1 [Reference] | 1.003 (0.943-1.066) | 1.108 (1.040-1.181) | - | - | 0.004 |
| Adjusted hazard ratios ^e^. | 1 [Reference] | 1.002 (0.941-1.067) | 1.100 (1.030-1.176) |  | - | 0.011 |
| **Fasting glucose** |  |  |  |  |  |  |
| Ranges (mg/dL) | < 70 | 70 – 99 | 100 – 125 | ≥ 126 | - |  |
| Alzheimer’s disease events | 41 | 3946 | 2226 | 777 |  |  |
| Person-years | 2963.7 | 323332.2 | 171620.7 | 53472.9 |  |  |
| Incidence ^a^. | 1383.4 | 1220.4 | 1297.0 | 1453.1 |  |  |
| Age-adjusted hazard ratios | 1 [Reference] | 0.893 (0.657-1.215) | 0.915 (0.672-1.246) | 1.033 (0.755-1.415) | - | 0.0006 |
| Adjusted hazard ratios ^f^. | 1 [Reference] | 0.932 (0.685-1.268) | 0.918 (0.674-1.250) | 0.891 (0.650-1.222) | - | 0.27 |
| **Systolic blood pressure** |  |  |  |  |  |  |
| Ranges (mmHg) | < 120 | 120 – 129 | 130 – 139 | ≥ 140 | - |  |
| Alzheimer’s disease events | 1556 | 1432 | 2064 | 1938 |  |  |
| Person-years | 118888.2 | 117361.4 | 167903.8 | 147236.0 |  |  |
| Incidence ^a^. | 1308.8 | 1220.2 | 1229.3 | 1316.3 |  |  |
| Age-adjusted hazard ratios | 1 [Reference] | 0.925 (0.861-0.993) | 0.887 (0.830-0.947) | 0.889 (0.831-0.95) | - | 0.001 |
| Adjusted hazard ratios ^g^. | 1 [Reference] | 0.884 (0.821-0.953) | 0.830 (0.772-0.893) | 0.800 (0.734-0.873) | - | <.0001 |
| **Diastolic blood pressure** |  |  |  |  |  |  |
| Ranges (mmHg) | < 80 | 80 – 89 | ≥ 90 | - | - |  |
| Alzheimer’s disease events | 3184 | 2641 | 1165 |  |  |  |
| Person-years | 258339.1 | 208254.6 | 84795.7 |  |  |  |
| Incidence | 1232.5 | 1268.2 | 1373.9 |  |  |  |
| Age-adjusted hazard ratios | 1 [Reference] | 1.000 (0.950-1.053) | 1.031 (0.964-1.103) | - | - | 0.451 |
| Adjusted hazard ratios ^h^. | 1 [Reference] | 1.046 (0.990-1.106) | 1.158 (1.066-1.259) | - | - | 0.001 |
| **Body Mass Index** |  |  |  |  |  |  |
| Ranges (kg/m^2^) | < 18.5 | 18.5 – 24.9 | 25 – 29.9 | 30 – 34.9 | < 35 |  |
| Alzheimer’s disease events | 378 | 4251 | 2107 | 245 | 9 |  |
| Person-years | 18155.5 | 316221.7 | 192177.6 | 24000.9 | 833.8 |  |
| Incidence | 2082.0 | 1344.3 | 1096.4 | 1020.8 | 1079.4 |  |
| Age-adjusted hazard ratios | 1 [Reference] | 0.952 (0.856-1.059) | 0.910 (0.814-1.017) | 0.891 (0.757-1.048) | 1.196 (0.617-2.318) | 0.474 |
| Adjusted hazard ratios ^h^. | 1 [Reference] | 0.929 (0.834-1.034) | 0.859 (0.765-0.964) | 0.828 (0.702-0.978) | 1.100 (0.567-2.135) | 0.265 |

Abbreviations: LDL-C, low-density lipoprotein cholesterol; HDL-C, high-density lipoprotein cholesterol

^a^. incidence was defined as events/100,000 person-years

^b^. Adjusted for sex, age, income, life styles (smoking, alcohol consumption and exercise), co-morbidities, health care visit frequency, medication history, body mass index, systolic/diastolic blood pressure, HDL-C, triglyceride, fasting glucose, and hemoglobin.

^c^. Adjusted for sex, age, income, life styles (smoking, alcohol consumption and exercise), co-morbidities, health care visit frequency, medication history, body mass index, systolic/diastolic blood pressure, HDL-C, triglyceride, fasting glucose, and hemoglobin.

^d^. Adjusted for sex, age, income, life styles (smoking, alcohol consumption and exercise), co-morbidities, health care visit frequency, medication history, body mass index, systolic/diastolic blood pressure, LDL-C, triglyceride, fasting glucose, and hemoglobin.

^e^. Adjusted for sex, age, income, life styles (smoking, alcohol consumption and exercise), co-morbidities, health care visit frequency, medication history, body mass index, systolic/diastolic blood pressure, LDL-C, HDL-C, fasting glucose, and hemoglobin.

^f^. Adjusted for sex, age, income, life styles (smoking, alcohol consumption and exercise), co-morbidities, health care visit frequency, medication history, body mass index, systolic/diastolic blood pressure, LDL-C, HDL-C, triglyceride, and hemoglobin.

^g^. Adjusted for sex, age, income, life styles (smoking, alcohol consumption and exercise), co-morbidities, health care visit frequency, medication history, body mass index, diastolic blood pressure, LDL-C, HDL-C, triglyceride, fasting glucose, and hemoglobin.

^h^. Adjusted for sex, age, income, life styles (smoking, alcohol consumption and exercise), co-morbidities, health care visit frequency, medication history, body mass index, systolic blood pressure, LDL-C, HDL-C, triglyceride, fasting glucose, and hemoglobin.

^i^. Adjusted for sex, age, income, life styles (smoking, alcohol consumption and exercise), co-morbidities, health care visit frequency, medication history, systolic/diastolic blood pressure, LDL-C, HDL-C, triglyceride, fasting glucose, and hemoglobin.

**Table S7. Stratified analysis according to medications prescription during the first 3 years of follow-up**^a.^.

|  | **Group 1** | **Group 2** | **Group 3** | **Group 4** | **Group 5** | **P for trend** | **Medication × group Interaction P** |
| --- | --- | --- | --- | --- | --- | --- | --- |
| Total cholesterol ^b^. |  |  |  |  |  |  | 0.663 |
| Without dyslipidemia medication (n=130743) | 1 [Reference] | 1.03 (0.978-1.084) | 1.053 (0.976-1.136) |  |  | 0.131 |  |
| With dyslipidemia medication (n=47843) | 1 [Reference] | 1.002 (0.916-1.095) | 1.072 (0.966-1.189) |  |  | 0.247 |  |
| LDL-C ^c^. |  |  |  |  |  |  | 0.846 |
| Without dyslipidemia medication (n=130743) | 1 [Reference] | 0.996 (0.942-1.054) | 1.030 (0.967-1.096) | 1.079 (0.987-1.179) | 1.056 (0.906-1.230) | 0.1 |  |
| With dyslipidemia medication (n=47843) | 1 [Reference] | 0.996 (0.905-1.095) | 1.020 (0.919-1.131) | 1.105 (0.975-1.253) | 1.039 (0.866-1.247) | 0.213 |  |
| HDL-C ^d^. |  |  |  |  |  |  | 0.069 |
| Without dyslipidemia medication (n=130743) | 1 [Reference] | 0.970 (0.920-1.022) | 0.934 (0.865-1.008) |  |  | 0.078 |  |
| With dyslipidemia medication (n=47843) | 1 [Reference] | 0.892 (0.820-0.970) | 0.965 (0.855-1.089) |  |  | 0.142 |  |
| Triglyceride ^e^. |  |  |  |  |  |  | 0.688 |
| Without dyslipidemia medication (n=130743) | 1 [Reference] | 1.027 (0.965-1.093) | 1.091 (1.020-1.168) |  |  | 0.013 |  |
| With dyslipidemia medication (n=47843) | 1 [Reference] | 1.015 (0.924-1.115) | 1.060 (0.962-1.169) |  |  | 0.258 |  |
| Fasting glucose ^f^. |  |  |  |  |  |  | 0.372 |
| Without diabetes medications (n=146765) | 1 [Reference] | 0.857 (0.660-1.113) | 0.850 (0.654-1.106) | 0.822 (0.621-1.088) |  | 0.336 |  |
| With diabetes medications (n=31821) | 1 [Reference] | 1.114 (0.628-1.977) | 1.012 (0.572-1.792) | 1.065 (0.602-1.885) |  | 0.868 |  |
| Systolic blood pressure ^g^. |  |  |  |  |  |  | 0.394 |
| Without antihypertensive medications (n=72607) | 1 [Reference] | 0.913 (0.833-1.001) | 0.851 (0.775-0.936) | 0.886 (0.786-0.998) |  | 0.049 |  |
| With antihypertensive medications (n=105979) | 1 [Reference] | 0.933 (0.862-1.009) | 0.851 (0.788-0.919) | 0.799 (0.732-0.873) |  | <0.0001 |  |
| Diastolic blood pressure ^h^. |  |  |  |  |  |  | 0.646 |
| Without antihypertensive medications (n=72607) | 1 [Reference] | 1.004 (0.932-1.082) | 1.074 (0.948-1.216) |  |  | 0.397 |  |
| With antihypertensive medications (n=105979) | 1 [Reference] | 1.058 (1.000-1.118) | 1.124 (1.036-1.220) |  |  | 0.004 |  |

Abbreviations: LDL-C, low-density lipoprotein cholesterol; HDL-C, high-density lipoprotein cholesterol

^a^. Without medication indicates that patients had not been prescribed medication within the first 3 years of follow-up; with medication indicates that they were prescribed medication within the first 3 years of follow-up.

^b^. Adjusted for sex, age, income, life styles (smoking, alcohol consumption and exercise), co-morbidities, health care visit frequency, medication history, body mass index, systolic/diastolic blood pressure, HDL-C, triglyceride, fasting glucose, and hemoglobin.

^c^. Adjusted for sex, age, income, life styles (smoking, alcohol consumption and exercise), co-morbidities, health care visit frequency, medication history, body mass index, systolic/diastolic blood pressure, HDL-C, triglyceride, fasting glucose, and hemoglobin.

^d^. Adjusted for sex, age, income, life styles (smoking, alcohol consumption and exercise), co-morbidities, health care visit frequency, medication history, body mass index, systolic/diastolic blood pressure, LDL-C, triglyceride, fasting glucose, and hemoglobin.

^e^. Adjusted for sex, age, income, life styles (smoking, alcohol consumption and exercise), co-morbidities, health care visit frequency, medication history, body mass index, systolic/diastolic blood pressure, LDL-C, HDL-C, fasting glucose, and hemoglobin.

^f^. Adjusted for sex, age, income, life styles (smoking, alcohol consumption and exercise), co-morbidities, health care visit frequency, medication history, body mass index, systolic/diastolic blood pressure, LDL-C, HDL-C, triglyceride, and hemoglobin.

^g^. Adjusted for sex, age, income, life styles (smoking, alcohol consumption and exercise), co-morbidities, health care visit frequency, medication history, body mass index, diastolic blood pressure, LDL-C, HDL-C, triglyceride, fasting glucose, and hemoglobin.

^h^. Adjusted for sex, age, income, life styles (smoking, alcohol consumption and exercise), co-morbidities, health care visit frequency, medication history, body mass index, systolic blood pressure, LDL-C, HDL-C, triglyceride, fasting glucose, and hemoglobin.

^i^. Adjusted for sex, age, income, life styles (smoking, alcohol consumption and exercise), co-morbidities, health care visit frequency, medication history, systolic/diastolic blood pressure, LDL-C, HDL-C, triglyceride, fasting glucose, and hemoglobin.
